# Poly(Vinyl Alcohol)-Saccharide Hydrogels with Size-Tunable Plasticization-to-Reinforcement for Flexible Sensors

**DOI:** 10.3390/gels12050375

**Published:** 2026-04-30

**Authors:** Guangyan Wang, Zhenzhen Wang, Shuqing Wei, Jianliang Bai, Cai Yan, Haigang Shi, Shaodong Li, Wenwei Lei

**Affiliations:** 1 Department of Chemistry, Changzhi University, Changzhi 046011, China; 24438427@czc.edu.cn (Z.W.); 24438428@czc.edu.cn (S.W.); czxybjl@163.com (J.B.); 12025919@czc.edu.cn (C.Y.); 12021810@czc.edu.cn (H.S.); lishaodong@czc.edu.cn (S.L.); 2 School of Environmental and Chemical Engineering, Yanshan University, Qinhuangdao 066004, China

**Keywords:** poly(vinyl alcohol) hydrogels, saccharides, tunable network, plasticization, physical interaction, strain sensing

## Abstract

This study demonstrates a molecular size-dependent strategy to regulate the network structure of poly(vinyl alcohol) (PVA) hydrogels using a series of saccharides with increasing molecular size—glucose, maltose, raffinose, soluble starch, and amylose. FTIR, XPS, XRD, and TG analyses reveal that increasing saccharide size shifts the network from plasticization to reinforcement, which is further confirmed by mechanical testing and rheological analysis. Small-molecule saccharides disrupt hydrogen bonds and enhance chain mobility, while macromolecular starches promote network regularity through strong hydrogen bonding and crystallization induction. This structural tunability ndows the resulting hydrogels with integrated functionalities: tensile strain increases from 640% to 1500%, self-healing efficiency reaches up to 90.6%, and high-fidelity electrocardiogram (ECG) signal acquisition is achieved with a signal-to-noise ratio of 39.84 dB, comparing favorably with commercial electrodes. This work establishes a structure–property relationship linking saccharide molecular size to network architecture and provides a versatile material platform for next-generation flexible wearable sensors and bioelectrodes.

## 1. Introduction

Hydrogels have found widespread applications in biomedical engineering [1,2], tissue engineering [3,4,5], energy storage [6,7], and wearable sensors [8,9,10,11] owing to their adjustable physicochemical characteristics. Poly(vinyl alcohol) (PVA) hydrogels stand out as promising materials, benefiting from high biocompatibility, excellent film-forming properties, and a high density of hydroxyl groups [12,13,14,15]. The functional properties of these hydrogels, such as mechanical behavior and self-healing capability, are intrinsically governed by their molecular network topology and interchain interactions, particularly hydrogen bonding. However, conventional PVA hydrogels suffer from limited mechanical diversity and inadequate self-healing capacity, which restrict their suitability for multifunctional flexible sensor applications [16].

To overcome these limitations, various strategies have been explored, such as incorporating functional fillers (e.g., carbon black, borax) and modulating network structures [17,18,19]. Among these approaches, saccharide molecules have recently emerged as green additives for regulating hydrogen bond networks in gel systems [20,21,22,23,24]. It has been reported that small-molecule saccharides (e.g., glucose) tend to disrupt intermolecular hydrogen bonds within polymer networks, exhibiting a plasticizing effect [21,25,26,27], while macromolecular saccharides (e.g., starch) interact with PVA backbones via strong hydrogen bonds, consequently enhancing the mechanical performance of materials [28,29,30,31]. Despite these qualitative observations, how the interaction mechanism transitions from plasticization to network reinforcement with increasing saccharide molecular size remains poorly understood, and systematic studies on the multiscale structural regulation of gel networks by different saccharides are still lacking, particularly in PVA systems.

In this work, we selected a series of saccharides with increasing molecular size—glucose, maltose, raffinose, soluble starch, and amylose—to investigate their influence on the structure and performance of PVA-based hydrogels. Our results reveal a clear trend: small-molecule saccharides predominantly act as plasticizers, disrupting PVA crystallization and enhancing chain mobility; medium-sized raffinose exhibits combined plasticizing and competitive hydrogen-bonding effects, introducing greater complexity into the hydrogen bond network; macromolecular starches promote network regularity through strong hydrogen bonding interactions and crystallization induction. This molecular size-dependent trend from plasticization toward network reinforcement provides a novel perspective for engineering PVA-based hydrogels. Leveraging this structural tunability, the resulting hydrogels demonstrate promising mechanical properties, self-healing capabilities, and sensing performance. With their integrated strain sensing, self-healing, and ECG monitoring functionalities, these PVA-saccharide hydrogels offer a valuable material platform for advanced flexible wearable electronics.

## 2. Results and Discussion

### 2.1. Preparation of PVA-Saccharide Hydrogels

**Figure 1:**
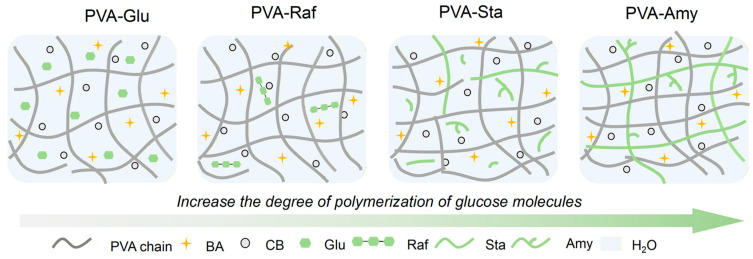
Schematic illustration of multiscale plasticization and chain entanglement induced by saccharides with varying degrees of polymerization.

To explore how different saccharides affect the network structure and hydrogen bonding of poly(vinyl alcohol) (PVA), a series of saccharides with varying degrees of polymerization were selected, including glucose (Glu), maltose (Mal), raffinose (Raf), starch soluble (Sta), and amylose (Amy). A 16.7% PVA solution containing carbon black (CB) was combined with a borax (BA) solution that contained different saccharides under continuous agitation. Following complete dissolution and removal of bubbles via centrifugation, the homogeneous solution was poured into a mold, cooled to −25 °C, then maintained at this temperature over 16 h. In this context, CB and BA were incorporated to further enhance network strength, while the addition of CB conferred favorable electrical conductivity and strain-sensing capabilities upon the hydrogel. Furthermore, the incorporation of different saccharides, including small-molecule saccharides, tends to interfere with the intermolecular hydrogen bonding among PVA chains, acting as network plasticizers. In contrast, macromolecular saccharides, owing to their longer chain structures, tend to establish robust hydrogen-bonding associations with the PVA matrix. Figure 1 illustrates the mechanism by which saccharides induce small-molecule plasticization and macromolecular reinforcement. The plasticization effect of small-molecule saccharides is characterized by the disruption of the PVA crystalline regions by small-molecule saccharides, which promotes chain slippage [20]. In contrast, macromolecular saccharides interact with PVA chains through strong hydrogen bonding, which effectively reinforces the network and improves mechanical properties.

**Figure 2:**
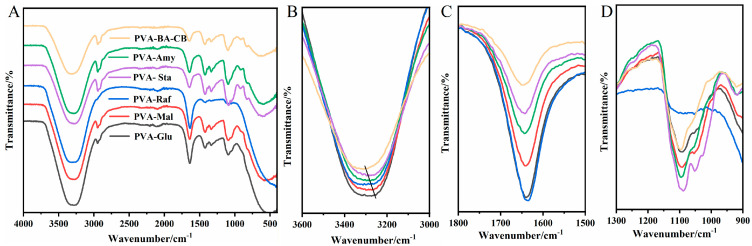
FTIR profiles of PVA and PVA-saccharide hydrogels. (**A**) Complete spectra (500–4000 cm^−1^). (**B**) O–H stretching region centered at ~3300 cm^−1^, showing saccharide size-dependent hydrogen bonding. (**C**) H–O–H bending region near 1640 cm^−1^. (**D**) α-1,4-glycosidic bond region (~1052 cm^−1^), indicating saccharide chain length.

Fourier transform infrared (FTIR) spectroscopy was used to investigate the chemical compositions and molecular-level interactions among a series of hydrogels (PVA, PVA-Glu, PVA-Mal, PVA-Raf, PVA-Sta, and PVA-Amy). Figure 2A shows that the characteristic peak of PVA-saccharide hydrogels at approximately 3300 cm^−1^ corresponds to the O–H stretching vibration [32]. As the degree of polymerization of the saccharides increases, the O–H band undergoes a blue shift relative to that observed for small-molecule saccharides, suggesting the formation of more intricate hydrogen bonding networks within the system (Figure 2B) [33]. The peak near 2900 cm^−1^ corresponds to the C-H stretching vibration [34,35]. Hydrogen bonds formed between raffinose and PVA strengthen intermolecular interactions, which restrict the vibration of -CH_2_- groups and lead to a reduction in peak intensity. The peak at 1640 cm^−1^ is attributed to the bending vibration of H–O–H bonds in water (Figure 2C) [33,36]. Starch formed stronger hydrogen bonds with water, immobilizing water molecules more firmly within the network, making it difficult for them to exhibit the vibrational characteristics of free water. For raffinose, the significantly enhanced peak intensity at this position originates from the generation of a structure that incorporates crystalline water. Soluble starch and amylose exhibited typical characteristic peaks of α-1,4-glycosidic bonds around 1052 cm^−1^ [37] (Figure 2D). The FTIR results revealed a progressive evolution of hydrogen bonding interactions with increasing saccharide molecular size. Small-molecule saccharides (glucose, maltose) primarily disrupt the original hydrogen bond network of PVA, while medium-sized raffinose introduces complex competitive hydrogen bonding and crystalline water. Macromolecular starches (soluble starch, amylose) establish strong hydrogen bonds with both PVA chains and water molecules, promoting network regularity. This molecular size-dependent transition in hydrogen bonding behavior provides direct spectroscopic evidence for the multiscale regulation of PVA network structures by saccharides.

**Figure 3:**
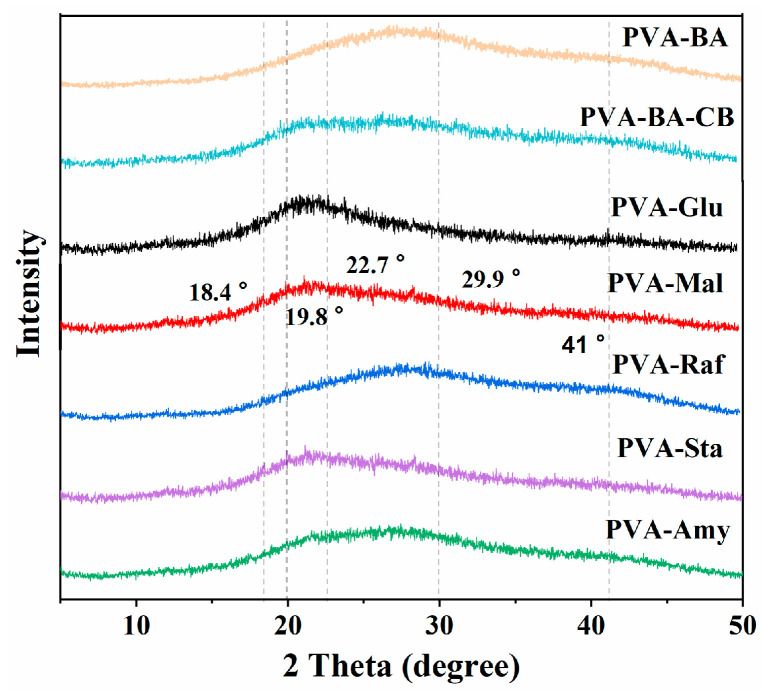
XRD patterns of PVA-saccharide hydrogels.

To examine how glucose addition affects hydrogen bonding, we performed additional X-ray diffraction (XRD), differential scanning calorimetry (DSC), and X-ray photoelectron spectroscopy (XPS) analyses. The crystallization behavior of the hydrogels was further analyzed using XRD. Figure 3 presents the XRD patterns of PVA, PVA-BA-CB, PVA-Glu, PVA-Mal, PVA-Raf, PVA-Sta, and PVA-Amy hydrogels. The diffraction profiles of these PVA-based systems are displayed in Figure 3. The PVA-BA hydrogel exhibits distinct diffraction peaks at 2θ = 19.8° and 22.7°, which are assigned to the (101) and (200) crystal planes of PVA, respectively [38,39,40]. In all samples, two broad halos centered at approximately 2θ = 28° and 41° are observed, consistent with the diffraction features of pure water [41]. Upon the incorporation of borax, the characteristic diffraction peaks of crystalline borax (typically observed at 2θ = 18.4° and 29.9°) [42] disappear, replaced by a broad halo, indicating that borax reacts with PVA to form an amorphous crosslinked network through boronate ester bonds. With the further addition of carbon black, the PVA crystalline peaks become broader and less intense, suggesting that carbon black disrupts PVA crystallization to some extent. The introduction of different saccharides further modulates the network structure, as reflected by the evolution of the diffraction patterns. The addition of small-molecule saccharides (glucose, maltose) or soluble starch significantly affects the borate ester crosslinking environment, leading to pronounced changes in peak shape. In contrast, the addition of raffinose introduces more complex hydrogen bonding interactions, which impose less disruption on the borate ester network, resulting in a diffraction profile similar to that of the carbon black-containing system. These results demonstrate that saccharide molecular size plays a critical role in regulating both the PVA crystalline structure and the borax-mediated crosslinking network.

**Figure 4:**
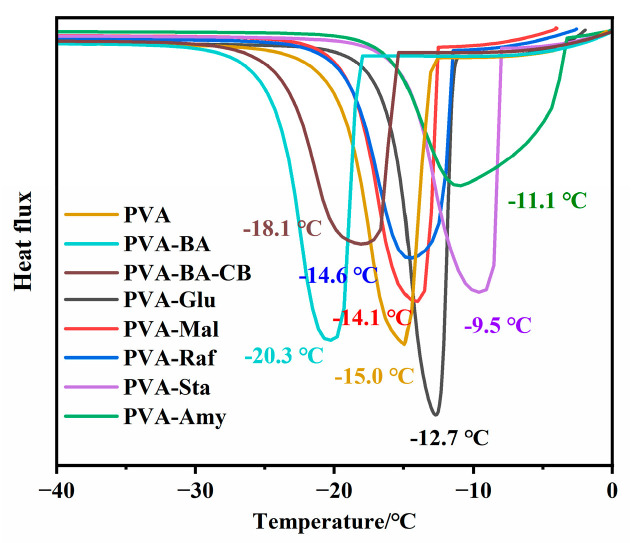
DSC curves of PVA-saccharide hydrogels.

To further investigate the effect of different saccharide additions on PVA hydrogen bonding, DSC analysis was performed. As shown in Figure 4, with increasing degree of polymerization of saccharides, the freezing point of the hydrogels exhibited a non-monotonic trend. Specifically, the freezing points of the PVA and PVA-saccharide hydrogels were −15.0 °C (PVA), −20.3 °C (PVA-BA), −18.1 °C (PVA-BA-CB), −12.7 °C (PVA-Glu), −14.1 °C (PVA-Mal), −14.6 °C (PVA-Raf), −9.5 °C (PVA-Sta), and −11.1 °C (PVA-Amy), respectively. Compared to pure PVA, the progressive freezing point depression upon BA addition was ascribed to the increased bound water content from dynamic borate ester crosslinking, which limited free water mobility and suppressed ice nucleation. Conversely, CB addition raised the freezing point by acting as a physical filler that disrupted the orderly packing of PVA chains and hindered the formation of PVA crystalline regions. More importantly, the PVA-saccharide hydrogels exhibit even higher freezing points compared to PVA-BA-CB. The introduction of small-molecule saccharides (such as glucose) disrupts the hydrogen bond network between PVA molecules, thereby enhancing chain segment mobility. This increased chain mobility suggests that a portion of free water becomes transformed into bound water, resulting in a reduced freezing point [21,43]. With increasing molecular weight of saccharides, the hydroxyl groups along the saccharide molecules establish competitive hydrogen bonds with PVA, further disrupting the original crystalline structure of PVA, leading to a continued decrease in freezing point. When long-chain macromolecular saccharides (such as starch and amylose) were introduced, they formed strong physical interactions with the PVA chain, which restricted molecular chain movement and caused the freezing point to rebound. Notably, macromolecular saccharides with different structures exhibited significant differences in their effects on PVA crystallization behavior. Amylose, due to its regular structure, can provide an ordered template for PVA chains, promoting PVA crystallization [44], with a freezing point of −11.1 °C, while soluble starch, with its branched structure leading to irregular molecular chain arrangement, was not conducive to inducing PVA crystallization, with a relatively higher freezing point of −9.5 °C.

**Figure 5:**
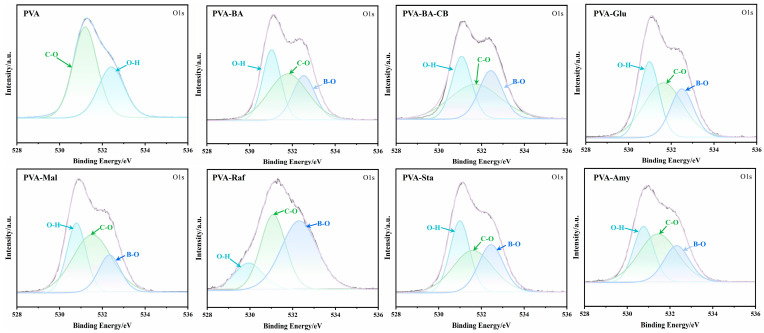
High-resolution XPS O1s spectra and deconvolution for PVA and PVA-saccharide hydrogels (the gray curve represents the raw data, and the purple curve represents the fitted results).

XPS analysis revealed the regulatory mechanism of saccharide molecular size on the PVA hydrogen bonding network at the electronic structure level, clearly presenting a transition pattern from “plasticization-dominated” to “physical interaction/crystallization-dominated” (Figure 5). Compared with pure PVA, the PVA-BA hydrogel exhibited an increased content of B–O bonds, and the O–H binding energy shifted from 532.4 eV down to 531.79 eV, which verifies the successful establishment of borate ester crosslinking between PVA and BA. Furthermore, upon the introduction of carbon black, no significant changes were observed in the XPS spectra. The addition of glucose resulted in the generation of a greater abundance of hydroxyl groups, thereby increasing the proportion of O–H. With increasing saccharide polymerization degree, the O1s binding energy showed a trend of first decreasing and then increasing, reflecting the evolution of the underlying interaction mechanism. Specifically, glucose, as a small-molecule saccharide, primarily exerts a plasticizing effect. Its penetration into the PVA network disrupts the original strong intermolecular hydrogen bonds, forming weak PVA-saccharide hydrogen bonds and a relatively low proportion of B-O bonds, manifested as the highest binding energy (O-H, 531.63 eV). As the polymerization degree increases, raffinose—a trisaccharide of medium molecular weight—exhibits dual effects of plasticization and competitive hydrogen bonding. Its numerous hydroxyl groups form a complex competitive hydrogen bond network with PVA, resulting in the lowest binding energy (O-H, 529.93 eV) and a high proportion of B-O bonds. The maximum full width at half maximum (FWHM) reflects the complexity of its oxygen chemical environment. When the saccharide further extends to long-chain starches, the interaction mechanism transitions to crystallization induction, as evidenced by the increased binding energy (O-H, 530.76 eV) and the sharp, symmetric O 1s peak shape.

**Figure 6:**
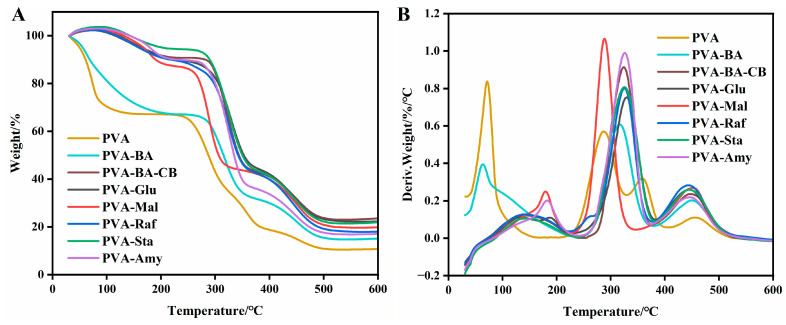
(**A**) TGA and (**B**) dTGA of PVA and PVA-saccharide hydrogels.

The thermal behavior of the hydrogel specimens was assessed via thermogravimetric (TGA) analysis, as illustrated in Figure 6A,B. Each sample displayed three distinct stages: water evaporation in the low-temperature region (0–150 °C), disruption of physical crosslinking in the medium-temperature region (150–250 °C), and main-chain degradation in the high-temperature region (250–600 °C) [45]. The onset decomposition temperatures (T_onset_) of PVA and PVA-BA were 58.01 °C and 51.35 °C, respectively. Upon the introduction of carbon black (CB), PVA-BA-CB exhibited a significantly higher T_onset_ of 104.97 °C, indicating that CB formed a stable crosslinking network with PVA, effectively immobilizing water molecules and enhancing thermal stability. In contrast, the addition of glucose (PVA-Glu) decreased T_onset_ to 93.58 °C, which was ascribed to the plasticizing action of glucose that disrupted the polymer matrix and promoted water loss. As the saccharide molecular size increased (e.g., from glucose to maltose), T_onset_ rose to 138.61 °C, suggesting that larger sugar molecules were less disruptive to the PVA network. Interestingly, raffinose (PVA-Raf) showed an anomalously low T_onset_ of 101.62 °C, which might be explained by the development of a tightly packed hydrogen-bonding network with PVA. This high crosslinking density compressed the free volume between polymer chains, making it easier for water molecules to be expelled from the network, thereby reducing thermal stability. With the introduction of long-chain molecules such as starch (PVA-Sta) and amylose (PVA-Amy), T_onset_ increased to 108.20 °C and 144.56 °C, respectively. This improvement was attributed to the formation of open, hydrophilic network structures that retained abundant water-binding sites while providing enhanced network integrity. These results confirmed that the transition from plasticization (by small molecules) to network reinforcement (by larger molecules) was accompanied by a significant improvement in thermal stability.

**Figure 7:**
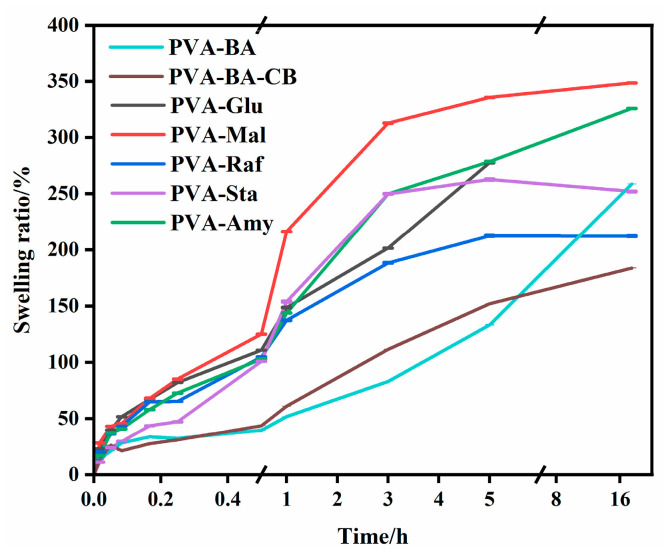
Swelling ratio of PVA and PVA-saccharide hydrogels.

To better understand how the addition of saccharides affects the material structure, we characterized the swelling behavior and microstructure of PVA hydrogels (without saccharides), and PVA-saccharide hydrogels were characterized. As presented in Figure 7, with the introduction of borax and carbon black, the crosslinked network structure of the hydrogels becomes increasingly dense. Consequently, as the immersion time increases, the swelling ratio of these hydrogels increases progressively. In contrast, upon the incorporation of saccharides, the hydrophilicity of the hydrogels increases due to the abundant hydroxyl groups introduced by the saccharides. During the initial immersion period (0–0.5 h), PVA-based composite hydrogels with different saccharide additions exhibited significantly different swelling behaviors. The PVA-Mal hydrogel showed a relatively high swelling ratio, while the PVA-Sta hydrogel exhibited a significantly lower swelling value. The increased swelling of the PVA-Mal system stems from two primary factors: firstly, compared with glucose, maltose has a larger molecular weight and diffuses more slowly, allowing it to remain longer in the gel matrix, thereby maintaining higher osmotic pressure that continuously drives water ingress; secondly, the plasticizing effect of maltose is more moderate than that of glucose, helping to maintain better network integrity and elasticity during swelling, thus supporting a higher equilibrium swelling ratio. In contrast, the initially low swelling of the PVA-Sta hydrogel originated from strong physical interactions between the soluble starch molecules and PVA polymer chains [44]. These interactions introduce additional physical crosslinking points into the network, substantially increasing its effective crosslinking density and elastic modulus. Consequently, water penetration at early stages must overcome considerable elastic restoring forces, resulting in a suppressed initial swelling rate. With prolonged immersion, the swelling ratio of the PVA-Mal hydrogel continued to increase steadily. Meanwhile, the PVA-Raf hydrogel maintained a consistently low swelling level throughout the test. Although raffinose is highly hydrophilic, its tendency to form microcrystalline domains or establish strong sugar–sugar and sugar–PVA hydrogen bonds creates supplementary physical crosslinks within the gel [46]. This elevation of network crosslinking density significantly constrains the extensibility of the polymer network, thereby limiting its overall swelling capacity.

**Figure 8:**
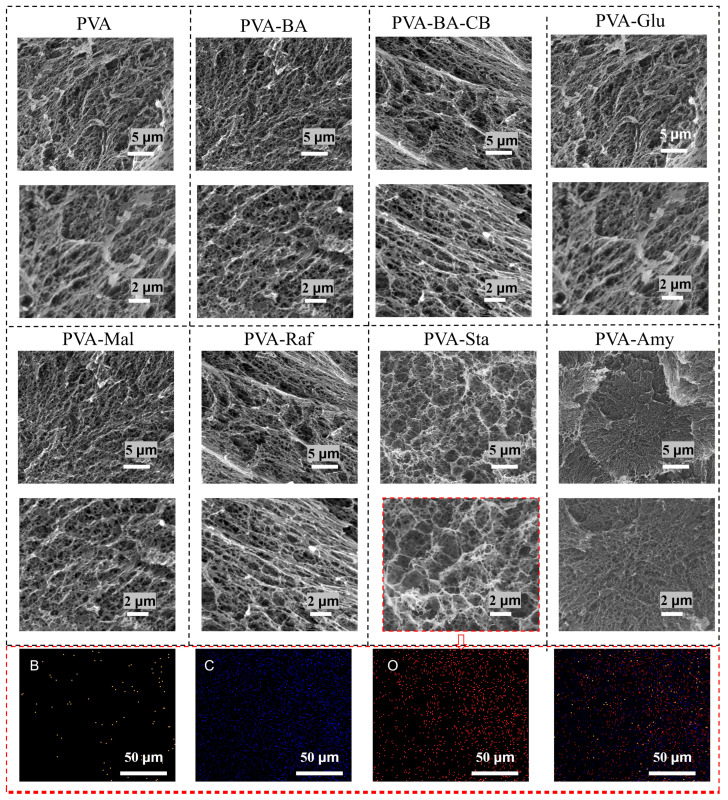
Morphological characterization of PVA and PVA-saccharide hydrogels obtained by scanning electron microscopy (SEM). SEM-EDX elemental mapping of PVA-Sta showing the distribution of B (orange), C (blue), and O (red).

Considering that the structure of hydrogels dried after swelling tests might be damaged, the hydrogels were directly immersed in water for 5 h of swelling and then freeze-dried to obtain their microstructure images (Figure 8). As shown in Figure 8, the introduction of borax and carbon black results in a denser network structure, leading to smaller pores. However, when the small-molecule sugar glucose was introduced, it disrupted the crystallization of PVA, resulting in a larger pore structure compared with the PVA-BA-CB hydrogel. Moreover, as the molecular size of the small-molecule sugar increased, the pore structure gradually became larger. With the introduction of long-chain starches, starch presented a larger honeycomb-like structure, while amylose exhibited a more dense pore structure [47]. This microstructural difference is highly consistent with the aforementioned swelling behavior and structural regulation mechanisms, further validating the regulatory effect of saccharide molecular size on the PVA network structure.

### 2.2. Mechanical Properties of PVA-Saccharide Hydrogels

**Figure 9:**
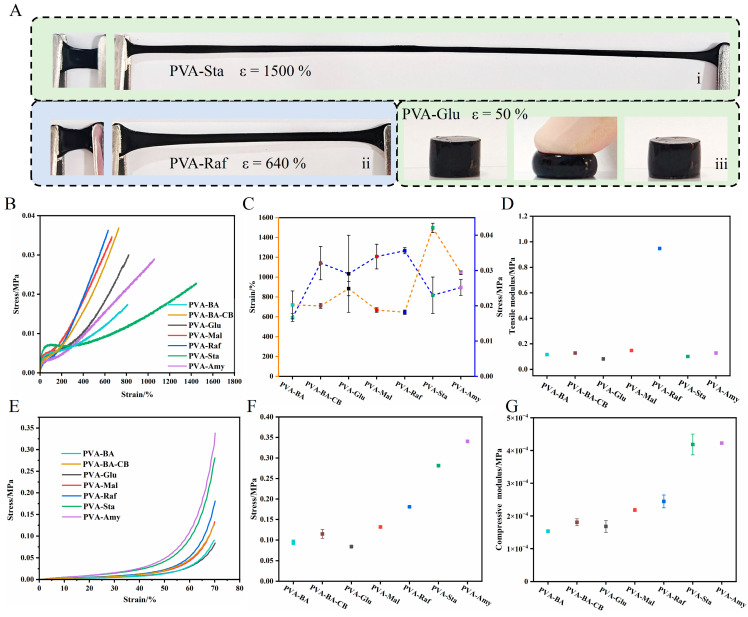
Mechanical performance of PVA-based hydrogels. (**A**) Photographic images illustrating mechanical behavior: (**i**) PVA-Glu under tension, (**ii**) PVA-Raf under tension, and (**iii**) PVA-Glu under compression. (**B**–**D**) Tensile properties: (**B**) representative stress–strain profiles, (**C**) tensile strength and elongation at break, and (**D**) Young’s modulus of the PVA-saccharide systems. (**E**–**G**) Compressive properties: (**E**) representative compressive stress–strain curves, (**F**) compressive stiffness, and (**G**) compressive modulus of the saccharide-modified hydrogels. (Cyan, PVA-BA; brown, PVA-BA-CB; black, PVA-Glu; red, PVA-Mal; blue, PVA-Raf; green, PVA-Sta; purple, PVA-Amy).

The mechanical performance of the hydrogel can be finely tuned by varying the degree of polymerization (DP) of the added sugar molecules. As illustrated in Figure 9A, this material exhibited high deformability, with stretchability exceeding 6.4 times for PVA-Raf and 15 times for PVA-Amy. Moreover, the PVA-Glu hydrogel could be compressed to half of its original thickness and then recover almost completely to its initial state. Figure 9B–D presents the tensile stress–strain profiles of pristine PVA and PVA-saccharide hydrogels. For tensile testing, PVA-BA exhibits a tensile strain of 719%, alongside a tensile strength of 0.017 MPa. Following the introduction of carbon black (PVA-BA-CB), the tensile strain decreases to 710% while the tensile strength increases to 0.032 MPa. As shown in Figure 9B, the stress–strain response features three distinct regimes. The initial linear rise in stress is due to strong hydrogen bonds that create a crosslinked network restricting polymer chain movement. Upon entering a plateau region, the carbohydrate additives serve as a lubricant, enabling molecular rearrangement through chain slippage under tension. This process ultimately leads to alignment of the polymer chains, resulting in a remarkably high elongation at break (up to 1497% for PVA-Sta). With increasing DP of small-molecule sugars, the plateau region in the stress–strain curve gradually narrows. Soluble starch, composed of fragments with a broad distribution of chain lengths, displays a wider plateau due to its polydisperse molecular weight. High-molecular-weight amylose (containing about 30% amylopectin) also retains a distinct plateau. Final elongation leads to full chain alignment and the formation of new hydrogen bonds, causing strain hardening until fracture. The results confirm the highly tunable mechanical behavior of these hydrogels.

Among small-molecule sugars, raising the degree of polymerization (DP) leads to an increase in tensile strength from 0.029 MPa to 0.035 MPa, whereas the elongation at break decreases from 884% to 646%. For the polysaccharides—soluble starch and amylose (30% amylopectin content)—the tensile strengths are comparable, but the elongation at break jumps significantly from 1045% to 1497% (Figure 9C). The higher tensile strength and lower elongation associated with increasing glucose DP mainly stem from glucose disrupting inter-chain hydrogen bonds within PVA, consequently restricting chain mobility. The introduction of medium molecular weight Raf into PVA creates a more complex hydrogen bonding network with higher crosslinking density, significantly increasing the material’s elastic modulus. Adding high-molecular-weight starch strengthens physical interactions between starch and PVA chains, which in turn enhances the hydrogel’s mechanical properties. In starch, while longer chains contribute to enhanced physical interactions, shorter fragments act as plasticizers, disrupting the polymer network and lowering the overall strength. The tensile modulus follows a trend similar to that of tensile strength (Figure 9D).

For compressive testing, the introduction of borax and carbon black similarly enhances the mechanical strength and compressive modulus of the hydrogels (Figure 9E). These results demonstrate that the introduction of borax and carbon black leads to a progressively denser crosslinked network structure, which enhances both tensile strength and compressive modulus at the expense of stretchability. In contrast to tensile behavior, compression testing revealed an opposite trend: both compressive strength and modulus increased with the DP of the sugar additives (Figure 9E–G). This divergence originates from the distinct roles sugars play under compression. Small-molecule sugars act as rigid fillers; as their DP rises, their supportive function becomes more effective. Similarly, for polysaccharides, the enhancement in strength stems from increased molecular weight and enhanced physical interactions within the PVA network.

**Figure 10:**
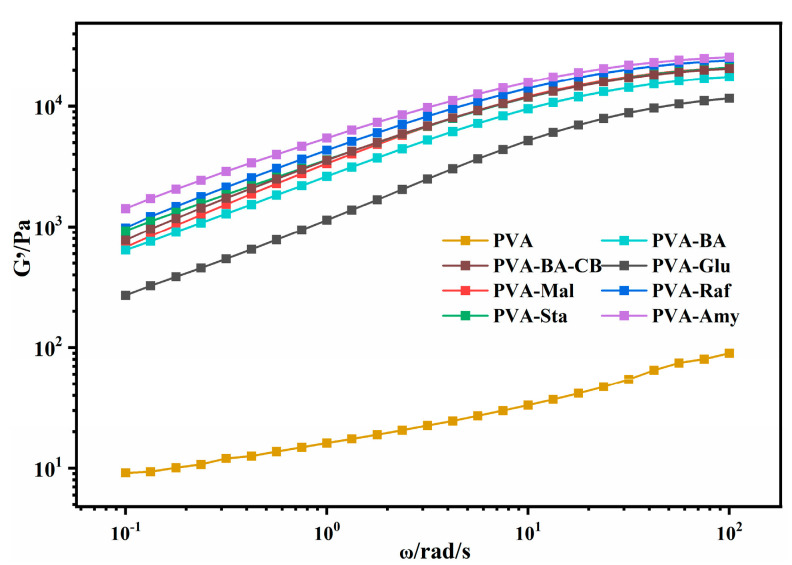
The rheological behavior of PVA and PVA-saccharide hydrogels was examined via frequency sweep tests.

As shown in Figure 10, the frequency sweep results reveal clear differences in viscoelastic behavior among the hydrogels. The PVA-BA and PVA-BA-carbon black hydrogels exhibit higher storage modulus (G′) values compared to pure PVA, confirming network reinforcement through borax crosslinking and carbon black filling. Glucose addition (PVA-Glu) reduces the storage modulus, consistent with a plasticizing effect. Interestingly, as the molecular size of the saccharide increases from maltose to starch, the storage modulus (G′) follows a non-monotonic trend—initially increasing for PVA-Raf, followed by a slight decrease for PVA-Sta, and ultimately increasing again for PVA-Amy. This non-monotonic behavior can be explained by three effects: the plasticizing effect of small-molecule sugars leads to a decrease in modulus; medium-molecular-weight sugars increase crosslinking density through multiple hydrogen bonds, thereby enhancing the modulus; and long-chain molecules (e.g., amylose) further strengthen the network stiffness through more extensive hydrogen bonding. Notably, this non-monotonic trend in modulus is consistent with the trend observed for tensile strength.

**Figure 11:**
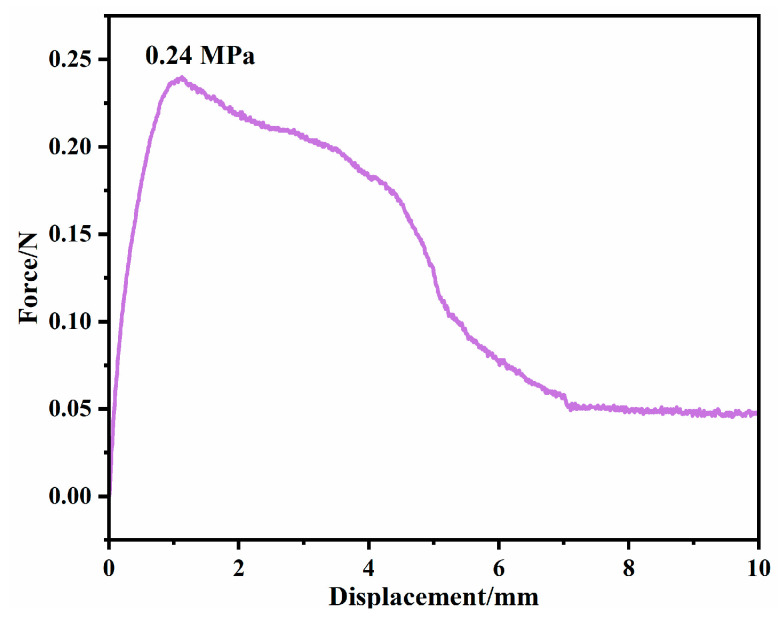
The rheological behavior of PVA and PVA-saccharide hydrogels was examined via frequency sweep tests (ω = 0.1–100 rad/s) at a fixed strain (γ) of 0.5% and a temperature of 25 °C.

To evaluate the adhesive behavior of the hydrogels, a tensile adhesion test was performed. As presented in Figure 11, the PVA-Sta hydrogel achieved an adhesion strength of 0.24 MPa against porcine skin. The observed adhesion capability stems from the plentiful hydroxyl moieties within starch, which facilitate interfacial hydrogen bonding interactions. This feature renders the PVA-polysaccharide hydrogel promising for potential applications on human skin.

### 2.3. Self-Healing Properties of PVA-Saccharide Hydrogels

**Figure 12:**
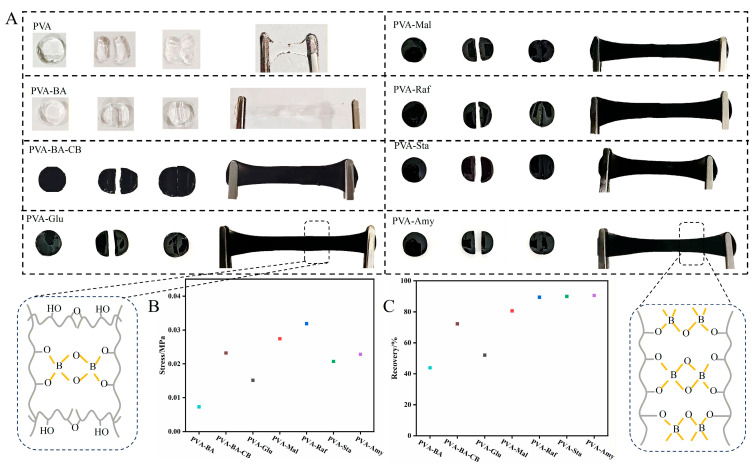
Self-healing performance of PVA and PVA-saccharide hydrogels. (**A**) Photographs demonstrating the self-healing process: cutting, contacting, and stretching of a healed hydrogel. (**B**) Tensile strain of in situ healed PVA-saccharide hydrogels. (**C**) Strain recovery efficiency of the in situ self-healing hydrogels.

The self-healing capability of the hydrogel, attributed to the synergistic effect of dynamic boronate ester bonds and hydrogen bonds [16,20], was quantitatively evaluated through visual observation and mechanical testing. To assess its autonomous healing behavior, two freshly cut hydrogel pieces were brought into contact under ambient conditions without any external stimulation. Specifically, a cylindrical hydrogel specimen containing boronate ester linkages was cut into two separate segments. Subsequently, these segments were simply rejoined by maintaining contact for 15 s at room temperature, as illustrated in Figure 12A. During tensile testing, the healed samples did not show any crack propagation and maintained mechanical integrity, indicating that structural continuity was fully restored through the reformation of dynamic bonds. The mechanical response of the PVA-saccharide hydrogels is presented in Figure 12B, highlighting their superior tensile performance. The self-healing efficiency was evaluated by applying Equation (1) to the stress at break data acquired from the stress–strain curves.

(1) $$Self-healing\;efficiency\;(\%)\;\;\;\;\;\;\;\;\;\;\;\;\;\;\;\;\;=\frac{Stress\;at\;break\;of\;healed\;sample}{Stress\;at\;break\;of\;prisine\;sample}\times 100\%$$

The self-healing behavior of PVA-BA and PVA-BA-CB hydrogels was systematically evaluated after only 15 s of healing. With the introduction of borax and carbon black, the crosslinked network structure becomes increasingly dense. Concurrently, the self-healing efficiency progressively increases (PVA-BA: 0.007 MPa, 43.87%; PVA-BA-CB: 0.023 MPa, 72.31%). These results demonstrate that the denser network structure—enabled by the dynamic borate ester crosslinks from borax and the physical filling effect of carbon black—facilitates more effective network reformation upon damage, thereby enhancing the self-healing capability of the hydrogels. Unlike the dense network formed by borax and carbon black, the introduction of small-molecule saccharides loosens the hydrogel network, leading to different mechanical and self-healing behaviors. After only 15 s of healing, the fracture stress of the PVA-saccharide hydrogels initially rose from 0.015 MPa to 0.032 MPa, then slightly dropped to 0.023 MPa as the saccharide changed from monosaccharide to trisaccharide and finally to amylose. The corresponding stress recovery efficiencies were 52.08%, 80.63%, 89.51%, 90.04%, and 90.63%, respectively (Figure 12C). The self-healing efficiency is governed by the dynamic interplay between boronate ester (B–O) crosslinking and the mobility of the dynamic network. As discussed in the XPS analysis, the proportion of B-O bonds varied significantly across different saccharide systems (Figure 5). The glucose system exhibited the lowest B-O bond content and lacked molecular entanglement, resulting in a self-healing efficiency of only 52.08%. As the molecular size of small saccharides increased, their disruptive effect on the PVA network diminished, leading to a higher B-O bond content and a corresponding increase in self-healing efficiency, which reached 89.51% for the Raf system. Upon the introduction of long-chain starches, the synergistic effect between B-O bonds and molecular entanglement further enhanced the self-healing efficiency, achieving the highest value of 90.63% for the amylose system.

**Figure 13:**
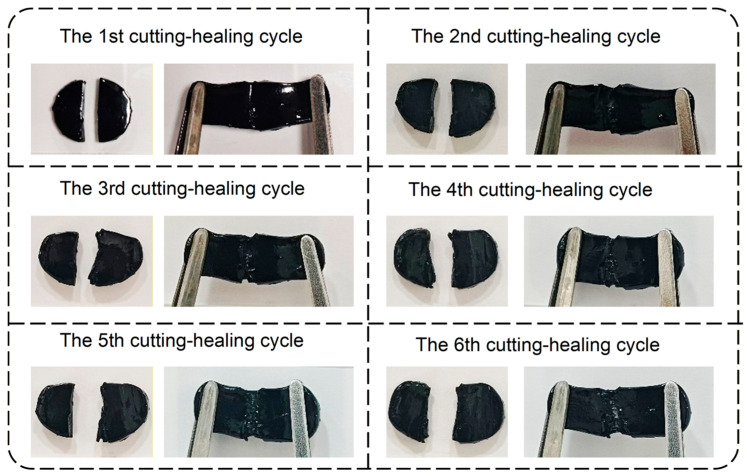
Self-healing performance of the PVA-Glu hydrogel over five consecutive cut–heal cycles.

To further verify the robustness of the self-healing behavior, repeated cycles of cutting and healing were performed on the same specimen. As presented in Figure 13, the PVA-Glu hydrogel was subjected to six successive cut–heal cycles. In each cycle, the hydrogel was completely cut into two pieces and then allowed to reconnect at room temperature for 15 s. These findings indicate that introducing saccharides of different molecular sizes can alter the bond types formed inside the network, which in turn dictates the ultimate self-healing performance of the resulting hydrogels.

### 2.4. Evaluation of Strain Sensing in PVA-Saccharide Hydrogels

Incorporating carbon black into the PVA-Sta hydrogel matrix greatly improved its electrical conductivity while preserving its favorable mechanical properties. This renders the composite a compelling candidate for constructing flexible, high-sensitivity strain sensors capable of accurately tracking various physiological signals in real time.

**Figure 14:**
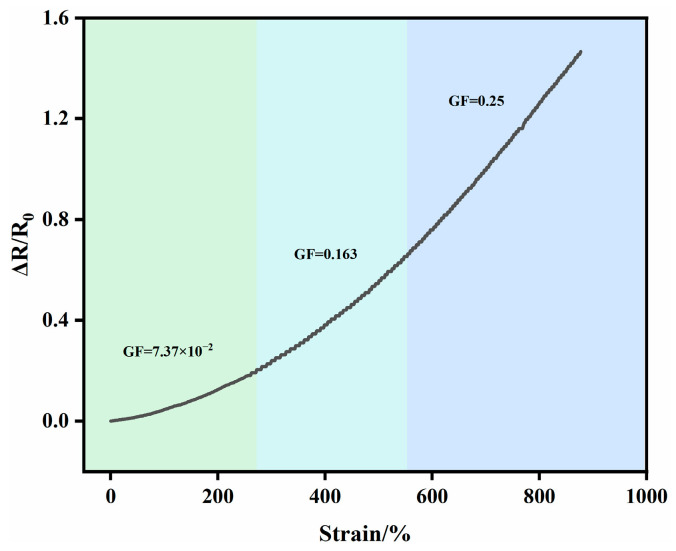
Relative resistance change versus strain plots.

The gauge factor (GF) is a key indicator for assessing the sensing sensitivity of PVA-Sta hydrogels. Figure 14 presents the GF values obtained under different strain intervals: 7.37 × 10^−2^ for 0–270% strain, 0.163 for 270–550% strain, and 0.25 for 550–870% strain. It should be noted that the gauge factor (GF) of the PVA-saccharide hydrogels was lower than that reported for many hydrogel-based strain sensors. This relatively low sensitivity was likely attributable to the trade-off between sensitivity and mechanical compliance. In our system, the focus was on achieving exceptional stretchability (exceeding 1500% strain) and durable self-healing performance (90.6% efficiency), which inherently limits the GF because highly compliant networks tend to exhibit smaller resistance changes upon deformation. Nevertheless, a moderate GF was often sufficient for monitoring large-scale human motions [48,49,50,51,52,53]. Thanks to its soft nature and rich hydrogen-bonding network, the hydrogel can conformally attach to the skin, allowing it to seamlessly follow body movements and effectively translate mechanical deformation into signal output (Figure 11) [54].

**Figure 15:**
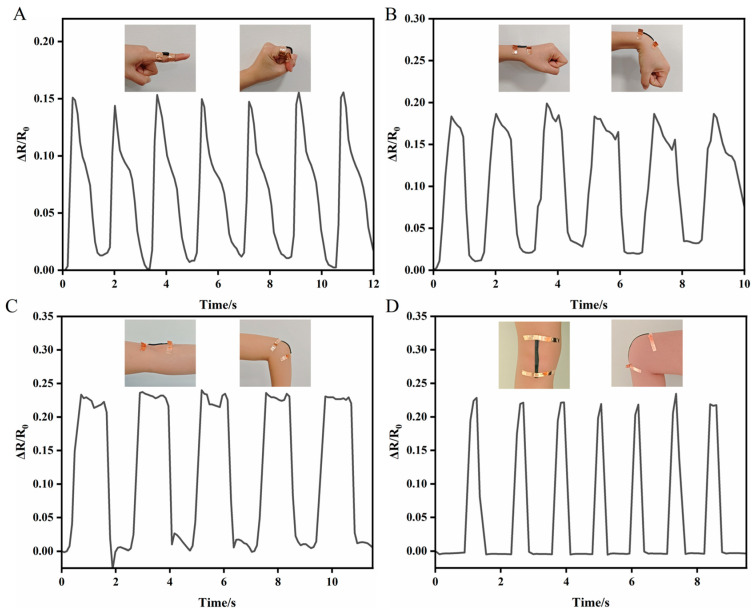
This figure illustrates the application of the PVA-Sta hydrogels in strain sensing. The instantaneous resistance variations in the hydrogel were recorded during the motion of different body joints, including the (**A**) finger, (**B**) wrist, (**C**) elbow, and (**D**) knee, demonstrating its capability as a flexible strain sensor.

The finger motion sensing ability was tested by mounting the sensor onto the index finger, while a Keithley 2400 Source Meter recorded the time-dependent ΔR/R0 throughout finger bending movements (Figure 15A). When the finger was bent to 0° and 90°, the ΔR/R_0_ value rose correspondingly and stayed steady while the finger remained fixed at each angle. After the finger was extended, ΔR/R_0_ completely recovered to its initial level, indicating that the hydrogel strain sensor can precisely differentiate various bending angles. The sensor was also applied to other body joints for broader motion tracking. During repetitive motions like wrist bending (Figure 15B), elbow flexion (Figure 15C), and knee bending (Figure 15D), the sensor consistently produced distinctive resistance increases that reverted to baseline once the joint was straightened. Notably, the relative resistance variations remained reproducible and consistent in peak magnitude across multiple cycles of joint movement.

### 2.5. ECG Monitoring Applications of PVA-Saccharide Hydrogels

**Figure 16:**
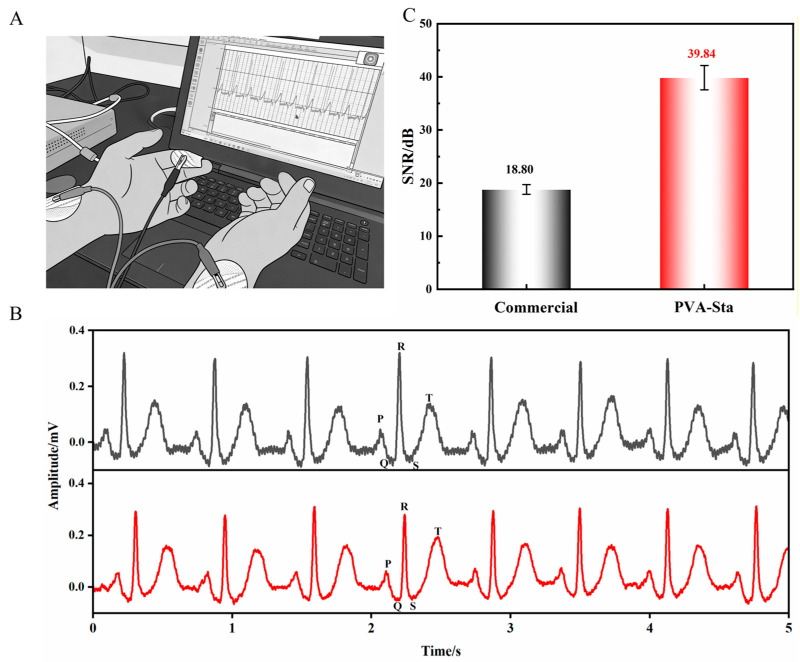
PVA-Sta hydrogels for bioelectrical monitoring. (**A**) ECG measurement setup schematic. (**B**) ECG signal comparison: commercial vs. PVA-Sta electrodes (P, Q, R, S, and T denote the characteristic waves of the electrocardiogram (ECG) signal). (**C**) SNR values of commercial and PVA-Sta electrodes.

The PVA-Sta hydrogel was rich in hydroxyl functional groups, which formed strong hydrogen bonds with polar moieties on the carbon black surface, thereby significantly enhancing interfacial adhesion. This characteristic made it particularly suitable for electrocardiogram (ECG) monitoring applications. As depicted in Figure 16A,B, we performed ECG measurements by placing commercial and PVA-Sta hydrogel electrodes on various joints of a volunteer. Both types of electrodes were able to record all major ECG waveforms with excellent signal fidelity: the P wave (atrial depolarization), QRS complex (ventricular activation/repolarization), and T wave (ventricular repolarization). Equations (2) and (3) were used to obtain the SNR of the ECG signals [55,56].

(2) $$SNR(voltage)={QRS}_{amp}/{Noise}_{amp}$$

(3) $$SNR(dB)=20{log}_{10}(SNR(voltage))$$

Here, QRS amplitude and noise level denote the magnitude of QRS peaks and the amplitude of the background signal excluding PQRST peaks, respectively. The calculated SNR values for commercial electrodes and PVA-Sta hydrogel electrodes are presented in Figure 16C. The commercial electrodes exhibited an SNR of 18.80 dB, while the PVA-Sta hydrogel electrodes achieved an SNR of 39.84 dB, showing a substantial difference between the two. These results unequivocally demonstrate that the PVA-Sta hydrogel, with its superior sensitivity and signal fidelity, represents an exceptional material platform for next-generation wearable health monitoring systems.

**Figure 17:**
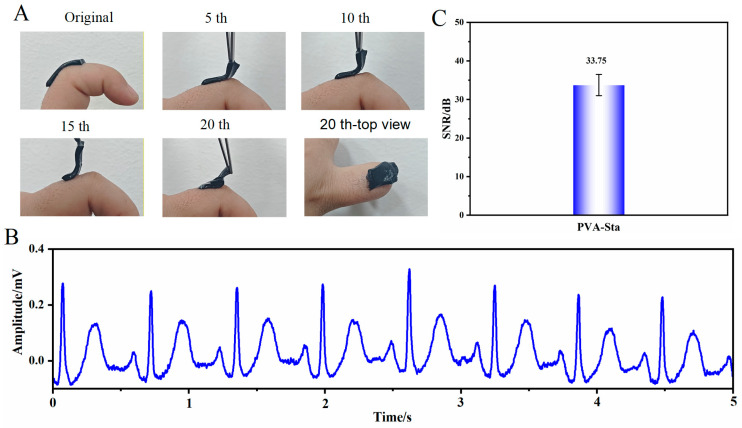
ECG monitoring performance of the PVA-Sta hydrogel electrode after multiple detachments. (**A**) Schematic illustration of the electrode undergoing 20 detachment cycles. (**B**,**C**) ECG recordings and corresponding signal-to-noise ratio (SNR) values obtained after 20 detachment cycles.

Mechanical durability tests were carried out to further evaluate the practicality of the hydrogel electrode. As shown in Figure 17, the PVA-Sta hydrogel electrode was repeatedly attached to and detached from human skin for 20 cycles, and its signal-to-noise ratio (SNR) was monitored throughout the test. The key finding is that after 20 cycles of skin attachment, the SNR remained at 33.75 dB. This result demonstrates that the PVA-saccharide hydrogel maintains excellent stability and durability even after prolonged repeated use.

## 3. Conclusions

In summary, based on qualitative trends obtained from structural characterizations, we propose a plausible mechanistic interpretation: as the molecular size of saccharides increases, their role may gradually transition from plasticization to network reinforcement. Specifically, small-molecule saccharides may primarily act as plasticizers, increasing the mobility of polymer chains; medium-sized saccharides may introduce more complex hydrogen bonding networks and serve as partial crosslinkers; and long-chain starches exhibit a tendency to form stronger hydrogen bonds and induce crystallization, thereby potentially contributing to network reinforcement.

Three key findings emerge from this study. First, as the molecular size of saccharides increases, the tensile strain of the hydrogels increases from 640% to 1500%. Second, the self-healing efficiency improves from 52.08% to 90.63%, showing a clear size-dependent trend. Third, the optimized PVA-saccharide hydrogel achieves a signal-to-noise ratio (SNR) of 39.84 dB, demonstrating excellent sensing capability. These quantitative observations are consistent with the proposed size-dependent transition from plasticization to network reinforcement. Based on these findings, we anticipate that this strategy of incorporating saccharides according to their molecular size may offer a useful approach for creating multifunctional hydrogels for biomedical and flexible electronics.

## 4. Materials and Methods

### 4.1. Materials

Polyvinyl alcohol 1799 (PVA-1799, 98–99% hydrolysis) was purchased from Shanghai Aladdin Biochemical Technology Co., Ltd. (Shanghai, China). Carbon black (CB) was purchased from Shanghai Titan Scientific Co., Ltd. (Shanghai, China). Glucose [D-(+) glucose monohydrate, Glu), analytical grade] and starch soluble (Sta, analytical grade) were purchased from Tianjin Aopu Sheng Chemical Co., Ltd. (Tianjin, China). D-Raffinose (Raf, purity: 99.0%) and amylose (Amy, amylose content greater than 70%, source: corn) were purchased from Shanghai Macklin Biochemical Co., Ltd. (Shanghai, China). Borax (Na_2_B_4_O_7_, BA, purity: 99.9%) was purchased from Anhui Senrise Technology Co., Ltd. (Anqing, China). Maltose monohydrate (Mal, purity: 99.0%) was purchased from Tianjin Kemiou Chemical Reagent Co., Ltd. (Tianjin, China). All reagents were used without further purification. Deionized water (18.2 MΩ·cm) from a Millipore purification system was used in all experiments.

### 4.2. Preparation of PVA Hydrogel

A blend consisting of 1.8 g of PVA and 0.03 g of carbon black in 9 mL of H_2_O was heated within a 90–100 °C water bath for 1 h with continuous agitation. Subsequently, 10 mL of 0.04 mol/L borax solution loaded with 1.25 wt% (relative to water) of various carbohydrates (Glu, Mal, Raf, Amy, and Sta) was added dropwise. The reaction mixture was then stirred and heated at 90–100 °C for 1 h. After centrifugal degassing, the resulting solution was cast into a mold of specified dimensions, frozen to −25 °C, and held at that temperature for 16 h.

### 4.3. Hydrogels Characterization

#### 4.3.1. Structural Characterization

Fourier transform infrared spectra (FTIR) of hydrogels were recorded in the wavelength range of 500–4000 cm^−1^ using an infrared spectrometer (Bruker ALPHA, Bruker optics, Ettlingen, Germany). The surface chemical composition of hydrogels was characterized using X-ray photoelectron spectroscopy (XPS, ESCALAB 250Xi, Thermo Scientific, Waltham, MA, USA). The freezing point was measured using a differential scanning calorimeter (DSC, TA DISCOVERY DSC2500, TA Instruments, New Castle, DE, USA). The cross-section morphology of hydrogels was evaluated by scanning electron microscopy (SEM) using a ZEISS EVO 10 (ZEISS, Jena, Germany). X-ray diffraction (PXRD) data were acquired using a Bruker D8 ADVANCE diffractometer equipped with Cu-Kα radiation (λ = 1.5406 Å). The diffraction patterns were recorded in the 2θ range of 5–50° with a scanning speed of 5° per minute. Thermogravimetric analysis was carried out on a TA Q500 (TA Instruments, New Castle, DE, USA) under a nitrogen atmosphere. The temperature was ramped from 30 °C to 600 °C at a constant rate of 20 °C/min.

#### 4.3.2. Swelling Measurements

The hydrogels were directly placed in water. At preset intervals, they were taken out, surface water was carefully removed by blotting with filter paper, and their weights were recorded.

#### 4.3.3. Mechanical Measurements

The mechanical properties of the hydrogels were measured with a universal testing machine (CMT2103, Shanxi Wanchen Testing Machine Co., Ltd., Jinan, China, 10 N and 100 N). Firstly, hydrogels were prepared in a size of 8 cm × 1 cm × 2 mm (rectangular, compression tests, 50 mm/min).

#### 4.3.4. Rheological Measurements

The rheological behaviors of hydrogel are characterized using a modular compact rheometer (Anton Paar, MCR 302). Cylindrical samples (15 mm diameter, 3 mm thickness) were prepared for testing. Frequency sweep tests were performed at a fixed strain (γ = 0.5%) over an angular frequency range (ω = 0.1–100 rad/s) at 25 °C.

#### 4.3.5. Adhesion Test

Porcine skin (obtained from a local market, cleaned, and prepared prior to use) was used to evaluate the adhesion strength of the hydrogels via a shear test. A 10 mm × 10 mm × 2 mm hydrogel specimen was sandwiched between two skin pieces, providing an overlap area of 1 cm^2^. The construct was subsequently subjected to tensile loading at a constant rate of 10 mm/min on a universal testing machine (e.g., CMT2103), and the peak force at failure was recorded. Equation 4 was used to calculate the adhesion strength, where σ represents the adhesion strength, F represents the maximum failure load of the bonded joint, and A represents the bonded area.

σ = F/A(4)

#### 4.3.6. Self-Healing Experiments

Hydrogel specimens (8 cm × 1 cm × 2 cm) were severed into two equal halves, and the detached fragments were then brought together. The healing was allowed to occur without any external stress or extra stimuli. Once the healing period was complete, tensile tests were re-conducted to measure the healing efficiency.

#### 4.3.7. Measurement of Strain Sensor Response Characteristics

The wearable sensor’s sensing ability was assessed by affixing it to distinct anatomical locations on the human body, i.e., the fingers, wrist, and knee. Variations in resistance induced by body motions were recorded utilizing a Keithley 2400 Source Meter. Equation (5) was employed to define the relative change in resistance, with R_r_ representing the resistance at any given moment and R_0_ signifying the initial resistance in the absence of applied strain. Subsequently, the gauge factor (GF) was determined from Equation (6), where ε corresponds to the strain applied.

ΔR/R_0_ = (R_r_ − R_0_)/R_0_ (5)

GF =(ΔR/R_0_)/*ε* (6)

## Data Availability

The original contributions presented in this study are included in the article; further inquiries can be directed to the corresponding author.
